# Genome-Wide Analysis Identifies *Rag1* and *Rag2* as Novel Notch1 Transcriptional Targets in Thymocytes

**DOI:** 10.3389/fcell.2021.703338

**Published:** 2021-07-12

**Authors:** Yang Dong, Hao Guo, Donghai Wang, Rongfu Tu, Guoliang Qing, Hudan Liu

**Affiliations:** ^1^Department of Hematology, Zhongnan Hospital of Wuhan University, Wuhan, China; ^2^Frontier Science Center for Immunology and Metabolism, Medical Research Institute, Wuhan University, Wuhan, China

**Keywords:** Notch1 dimerization, Recombination activating genes, T-cell acute lymphoblastic leukemia, Double negative thymocyte, T-cell development

## Abstract

Recombination activating genes 1 (*Rag1*) and *Rag2* are expressed in immature lymphocytes and essential for generating the vast repertoire of antigen receptors. Yet, the mechanisms governing the transcription of *Rag1* and *Rag2* remain to be fully determined, particularly in thymocytes. Combining cDNA microarray and ChIP-seq analysis, we identify *Rag1* and *Rag2* as novel Notch1 transcriptional targets in acute T-cell lymphoblastic leukemia (T-ALL) cells. We further demonstrate that Notch1 transcriptional complexes directly bind the *Rag1* and *Rag2* locus in not only T-ALL but also primary double negative (DN) T-cell progenitors. Specifically, dimeric Notch1 transcriptional complexes activate *Rag1* and *Rag2* through a novel *cis*-element bearing a sequence-paired site (SPS). In T-ALL and DN cells, dimerization-defective Notch1 causes compromised *Rag1* and *Rag2* expression; conversely, dimerization-competent Notch1 achieves optimal upregulation of both. Collectively, these results reveal Notch1 dimerization-mediated transcription as one of the mechanisms for activating *Rag1* and *Rag2* expression in both primary and transformed thymocytes. Our data suggest a new role of Notch1 dimerization in compelling efficient TCRβ rearrangements in DN progenitors during T-cell development.

## Introduction

Notch receptors are a family of heterodimeric transmembrane proteins that determine cell fate decisions and growth control. A prime example is that Notch1, one of the four isoforms (Notch1–4), plays an essential role in T-cell development and homeostasis. From a surface protein to a nuclear transcription factor, Notch1 signaling becomes active upon interaction with the Delta and Serrate family of ligands (Delta-like 1, 3, and 4; and Jagged 1 and 2) on juxtaposed cells. This interaction triggers conformational changes of Notch1, allows stepwise proteolytic cleavages, and releases intracellular Notch1 (ICN1). ICN1 then rapidly translocates into the nucleus and forms a transcriptional activation complex with the DNA binding factor RBPJ (also known as CSL) and coactivator of the mastermind family (MAML) (Kopan and Ilagan, 2009). As previously reported, ICN1/RBPJ/MAML complexes dimerize in the nucleus through cooperatively assembling on a specific sequence-paired site (SPS) in the promoter/enhancer of target genes, where the ANK domain within ICN1 mediates the dimer formation (Nam et al., 2007; Arnett et al., 2010).

Notch1 serves as a key regulator required at multiple stages for T-cell progenitor specification and maturation. Notch1 signaling determines T-cell lineage commitment and is essential to generate the early T-cell progenitors (ETP) from multipotent lymphoid progenitors (Sambandam et al., 2005; Tan et al., 2005). Inactivation of Notch1 signaling in hematopoietic progenitors results in a complete failure of T-cell generation, while it induces ectopic B-cell development in the thymus (Pui et al., 1999). Conversely, Notch1 overexpression causes extrathymic T-cell development in bone marrow at the expense of B cells (Radtke et al., 1999). Continuous Notch1 signaling is imperative in lymphocyte developmental progression through ETP, double negative 2 (DN2) and DN3 stages until completion of β-selection, a critical checkpoint where thymocytes successfully assemble pre-T-cell receptors (pre-TCRs) composed of a pre-T-cell antigen receptor α (pTα) chain and a functional TCRβ chain (Radtke et al., 2010). Notch1 expression drastically decreases after β-selection when thymocytes advance to double positive (DP) stage. During the progression, Notch1 provides essential differentiation, proliferation, survival, and metabolic signals, mainly *via* inducing the expression of downstream targets such as *Tcf1* (Weber et al., 2011), *Ptcra* (Reizis and Leder, 2002), *Myc* (Weng et al., 2006), *Il7r* (Gonzales-Garcia et al., 2009), and *Hes1* (Tomita et al., 1999; Kaneta et al., 2000). Notch1 dimerization appears essential in driving DN3 thymocytes through the β-selection, in part due to its ability to activate dimer-dependent target genes *Ptcra* and *Myc* (Liu et al., 2010).

When aberrantly activated, Notch acts as an oncogene driving acute T-cell lymphoblastic leukemia (T-ALL) (Aster et al., 2011; Liu et al., 2011; Paganin and Ferrando, 2011). Constitutive Notch1 expression in murine hematopoietic progenitors uniformly causes T-ALL (Pear et al., 1996). Moreover, the fact that more than half of the T-ALL patient samples bear gain-of-function Notch1 mutations suggests a central role of Notch1 in leukemogenesis (Weng et al., 2004). Consequently, enhanced Notch1 activity activates a spectrum of downstream targets that mediate leukemogenesis including *Myc* (Weng et al., 2006), *Hes1* (Real et al., 2009), *Lef1* (Spaulding et al., 2007), and *Deptor* (Hu et al., 2017). Unlike wild-type (WT) Notch1, dimer-disrupted allele failed to induce T-ALL in the mouse model, presumably due to the inactivation of dimer-responsive target *Myc* (Liu et al., 2010). It remains to be determined any additional Notch1 targets sensitive to dimeric Notch1 signaling.

*Rag1* and *Rag2* encode site-specific endonucleases to create double-stranded breaks (DSB) in the process of V(D)J recombination to constitute the diverse repertoire of T-cell receptors and B-cell immunoglobulin (Ig) receptors in response to diverse exogenous pathogens (Helmink and Sleckman, 2012; Miyazaki et al., 2020). Mice deficient in either *Rag1* or *Rag2* expression are blocked in T- and B-cell differentiation and exhibit a severe combined immunodeficiency (SCID) phenotype (Mombaerts et al., 1992; Shinkai et al., 1992). Expression of *Rag1* and *Rag2* is tightly controlled through a variety of DNA *cis*-elements and *trans*-factors, frequently in a T- or B-cell-specific manner (Kuo and Schlissel, 2009). Aberrant *Rag1*/2 activity is linked to oncogenic translocations associated with immature human and mouse B and T leukemia/lymphoma (Zhang et al., 2010).

In the present study, we identified *Rag1* and *Rag2* as Notch1 downstream targets from the gene expression profiling analysis in murine T-ALL T6E cells. Further mechanistic analyses reveal that ICN1 directly binds the *Rag2* locus to activate transcription, where we uncover a novel *cis*-element specifically recognized by the dimeric ICN1/RBPJ/MAML1 complexes. The Notch1-dimer dependency is not only restricted to T-ALL cells but also observed in DN progenitors, suggesting a new role of Notch1 in the developmental stage prior to β-selection−advancing TCRβ rearrangement. We thereby present a novel mechanism of Notch1-mediated transcriptional activation of *Rag1* and *Rag2*, involved in both T-cell development and leukemia.

## Results

### Recombination Activation Gene 1 and 2 Are Regulated by Dimeric Notch Signaling

**FIGURE 1:**
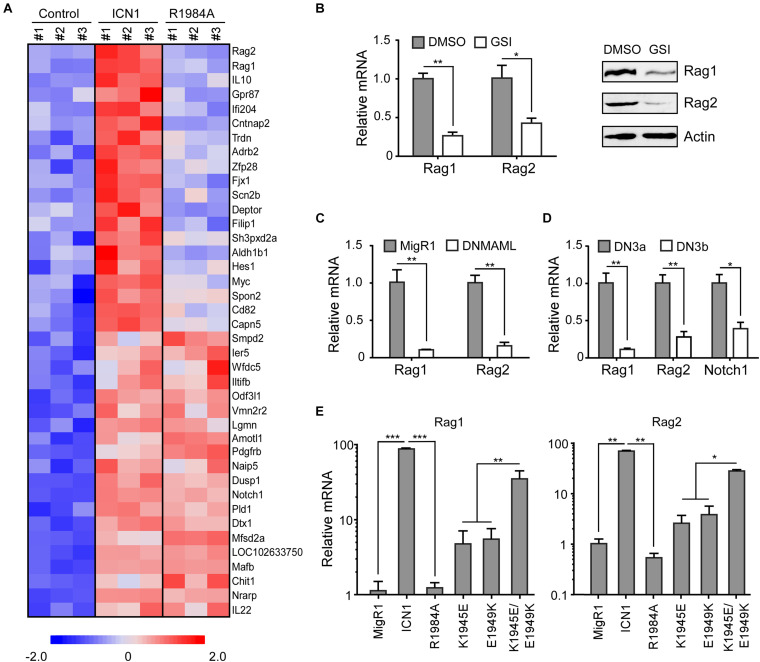
Notch signaling induces *Rag1* and *Rag2* expression. **(A)** Identification of Notch1 dimer-dependent target genes. Notch1-dependent genes were determined from a microarray analysis in murine T-ALL T6E cells (GSE97465). Each gene expression is displayed with colored boxes which are scaled in the bottom. ICN1 expression means Notch1 monomer and dimer activation, and R1984A expression denotes only Notch1 monomer activation. Expression data from 20 genes most strictly dependent on the Notch1 dimer and 20 genes equally induced by both Notch1 dimer and monomer are depicted (*p* < 0.05). **(B)** T6E cells were treated with JC-18 (1 μM) or DMSO for 24 h. *Rag1* and *Rag2* mRNA and proteins were detected by real-time PCR (left) and immunoblots (right). **(C)** T6E cells were transduced with DNMAML1 or empty vector MigR1 surrogated with a GFP marker for 24 h. GFP^+^ cells were sorted and subjected to mRNA analysis by real-time PCR. **(D)** DN3a and DN3b cells were isolated from C57BL/6 mice. *Rag1* and *Rag2* mRNA, along with positive control *Notch1* mRNA, were evaluated by real-time PCR. **(E)** T6E cells were infected by retroviruses expressing MigR1, WT ICN1, or designated dimerization mutants, then subjected to JC-18 treatment for 24 h. *Rag1* and *Rag2* mRNA levels were examined by real-time PCR. Above all, the expression of *Rag1* and *Rag2* relative to 18S RNA is shown as the mean of values from triplicate wells ± SD. Representative data from three independent experiments are shown. ***p < 0.001, **p < 0.01, and *p < 0.05.

Although the dimerization of Notch1 transcriptional complex is required for T-cell development and leukemogenesis (Liu et al., 2010; Severson et al., 2017), identities and the functional importance of Notch1 dimer-responsive targets remain largely unknown. To disclose novel dimeric Notch1 targets, we reanalyzed a genome-wide gene expression profiling (GSE97465) in a Notch1-dependent murine T-ALL cell line T6E (Severson et al., 2017). In this microarray data, T6E cells were infected with retroviruses expressing empty vector MigR1 with a surrogated GFP marker, MigR1 containing constitutive WT intracellular Notch1 (ICN1) or a dimerization-disrupted mutant ICN1 R1984A (R1984A) (Liu et al., 2010). Candidate genes which were efficiently induced by WT ICN1 but not ICN1 R1984A caught our attention. Filtered gene sets were aligned with fold changes to generate the heatmap of differential genes in response to Notch1 dimer and monomer (Figure 1A). While ICN1 R1984A upregulated many known Notch1 targets, including *Dtx1* and *Nrarp*, it failed to turn on a member of dimer-dependent genes, including previously reported *Myc*, *Hes1*, and *Deptor* (Liu et al., 2010; Hu et al., 2017). Of particular note, *Rag1* and *Rag2* rank at the top to be most differentially regulated by ICN1 and R1984A (Figure 1A).

Since ICN1 dramatically increased the expression of *Rag1* and *Rag2* as compared with the vector alone, we first confirmed whether their expression depends on Notch1 by analyzing the mRNA levels in various Notch1 inhibitory scenarios. γ-Secreatse inhibitor (GSI) JC-18 treatment, which suppresses Notch activation, in T6E cells resulted in decreased mRNA abundance of *Rag1* and *Rag2* (Figure 1B, left). Consistently, we detected lower *Rag1* and *Rag2* protein levels in T6E cells upon Notch inhibition (Figure 1B, right). As a further support, GSI treatment in human T-ALL KOPTK1 and CUTLL1 cells resulted in a significant decrease in mRNA and protein levels of *RAG1* and *RAG2* (Supplementary Figures 1A,B). We next expressed a dominant negative MAML1 (DNMAML1) to specifically repress the Notch1 pathway and observed sharp decreases of *Rag1* and *Rag2* as well (Figure 1C). Using primary murine thymocytes, we then investigated a physiological condition whereby Notch1 is downregulated. At the checkpoint of T-cell receptor β-selection, DN3a cells differentiate to DN3b cells, while Notch1 undergoes a dramatic decline upon the completion of TCRβ rearrangement. Correspondingly, *Rag1* and *Rag2* displayed similar reductions at the transition of DN3a to DN3b (Figure 1D).

The microarray data showed that both *Rag1* and *Rag2* were activated by ICN1 but not by dimerization-disrupted mutants R1984A (Figure 1A). To validate these data, we took advantage of various ICN1 mutants that are involved in the dimer formation of ICN1/RBPJ/MAML1 complexes on DNA, scoring their effects on *Rag1* and *Rag2* expression in T6E cells (Liu et al., 2010). Retroviruses expressing empty vector MigR1, or MigR1-ICN1, R1984A, K1945E, E1949K, and K1945E/E1949K were infected to T6E cells, followed by 24 h JC-18 treatment to repress endogenous Notch1 activity. GFP^+^ cells were sorted to analyze *Rag1* and *Rag2* mRNA abundance. Consistent with the microarray data, both *Rag1* and *Rag2* were activated by ICN1 but not by dimerization-disrupted mutant R1984A (Figure 1E). Expression of K1945E or E1949K alone, which breaks the intermolecular salt bridge between Notch1 dimer (Nam et al., 2007), induced moderate *Rag1* and *Rag2* expression. Notably, the double mutant K1945E/E1949K, which restores electrostatic complementarity and thereby the critical salt bridge essential for dimer formation (Nam et al., 2007), reconstituted the WT ICN1 activity to stimulate the *Rag1*/*2* mRNA expression (Figure 1E). Interestingly, we noticed that WT ICN1 but not dimerization-disrupted mutant R1984A was capable of activating the *RAG1/2* mRNA expression in human KOPTK1 and Jurkat cells (Supplementary Figure 1B). Collectively, these data argue that Notch1 dimerization-mediated transcriptional activation is a prominent and conserved mechanism underlying *Rag1* and *Rag2* regulation in T-ALL cells.

### Notch1 Directly Binds the Transcriptional Regulatory Region of *Rag2* Gene

**FIGURE 2:**
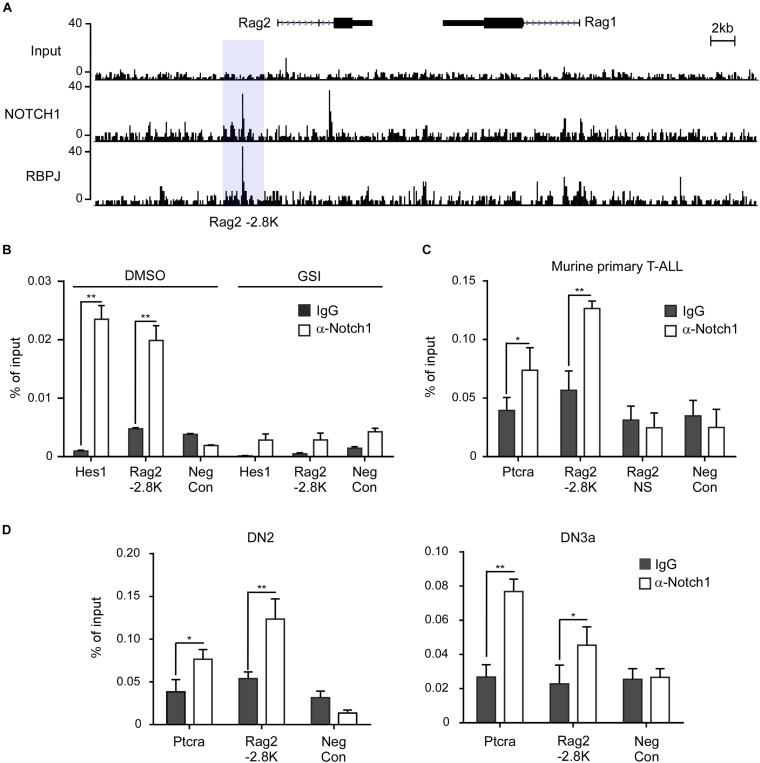
Notch1 directly binds the *Rag*1/2 locus in T-ALL cells and T-cell progenitors. **(A)** Analysis of the *Rag2* locus in the ChIP-seq data (GSE 29600). The binding signals of Notch1 and RBPJ on the -2.8 kb site upstream of the *Rag2* gene are highlighted in light gray. **(B–D)** Chromatin immunoprecipitation (ChIP) was performed on cross-linked fragmented DNAs prepared from T6E cells treated with DMSO or 1 μM JC-18 for 24 h **(B)**, murine primary T-ALL samples **(C)**, or murine DN2 and DN3a cells **(D)**. Eluted DNAs were then analyzed by qPCR using primers flanking the -2.8 kb site. The amount of DNA amplified from immunoprecipitated DNAs was normalized to that amplified from input DNA. Each sample was prepared from at least two independent experiments and run in triplicates. *Rag2* NS, non-specific sequence in *Rag2* locus; Neg Con, non-specific sequence in the *Nanog* gene used as the negative control. **p < 0.01 and *p < 0.05.

To determine if Notch1 transcriptional complex directly binds to and activates the gene expression, we first inspected the *Rag1*/*2* locus from the chromatin immunoprecipitation sequencing (ChIP-seq) data which provide comprehensive Notch1 and RBPJ binding sites in the genome of T6E cells (Wang et al., 2011). Within the *Rag* locus where *Rag1* and *Rag2* are juxtaposed with opposite orientation, we detected strong signals of Notch1 and RBPJ binding in the upstream 5′ region of the *Rag2* gene (Figure 2A). The tentative binding site locates at ∼2.8 kb from the transcription initiation site of the *Rag2* gene and contained a previously characterized *Rag2* P4 enhancer (Monroe et al., 1999; Wei et al., 2002). We reasoned that this enhancer region may include key *cis*-elements responsible for the *Rag2* transcription (Figure 2A).

ChIP assays were performed to validate Notch1 localization at the 5′ site upstream of *Rag2* in T-ALL cells. In T6E cells, Notch1 was found to bind to the *Rag2* -2.8 kb, like the well-characterized Notch1 binding site in the *Hes1* promoter. DNA regions without RBPJ binding site were included as a negative control (Figure 2B). By inhibiting γ-secretase-mediated proteolytic cleavage of membranous Notch1, GSI treatment decreased the nuclear ICN1 abundance, thus reducing global occupancy of Notch1/RBPJ binding sites in the genome, including that at the *Rag2* -2.8 kb site as well as the *Hes1* promoter. We further examined the Notch1 occupancy in primary T-ALL cells derived from the *Lck*-Cre Kras^G12D^ transgenic mice (Chiang et al., 2008). *Ptcra*, a Notch1 direct target gene encoding the pre-T-cell antigen receptor α (Reizis and Leder, 2002; Liu et al., 2010), was bound by Notch1 at its enhancer region. Similarly, Notch1 bound the *Rag2* -2.8 kb site but not a non-specific region in the *Rag2* locus, nor a *Rag2* irrelevant DNA region bearing no RBPJ binding site shown as a negative control (Figure 2C).

When early T-cell progenitors migrate into the thymus, undergoing stepwise differentiation to mature T cells, *Rag1* and *Rag2* mRNA start to increase for TCRβ rearrangement. Their levels reach the peak at DN3a stage right prior to β-selection. Whether Notch1 plays a role to induce *Rag1* and *Rag2* at this developmental stage is not yet clarified. We examined the occupancy of Notch1 on the identified -2.8 kb site in the sorted DN2 and DN3a cells from C57BL/6 mice (Figure 2D). ChIP assays revealed the Notch1 binding at the *Rag2* -2.8 kb in both DN2 and DN3a cells, similar to the previously characterized target gene *Ptcra*. Besides Notch1, RBPJ also occupied the same region (data not shown). In contrast, DNA sequences without RBPJ binding site, as the negative control, were not bound by either Notch1 or RBPJ. Taken together, these results provide strong evidence that Notch1 directly and specifically binds to the regulatory region upstream of *Rag2*.

We also searched the tentative binding sites of Notch1 and RBPJ on the *RAG1/2* locus in human T-ALL cells from previously reported ChIP-seq data (Severson et al., 2017). It appears that the conserved binding sites around -2.8 kb of the *Rag2* gene are not present in CUTLL1 cells (Supplementary Figure 2A). Instead, a prominent distal Notch1 binding signal, which was associated with the RBPJ and H3K27ac binding peaks, was detected at 87 kb upstream of the transcription initiation site of the *RAG2*. Of note, GSI treatment reduced the binding signal intensity of Notch1 and RBPJ. This Notch1 and, mostly likely, *RAG2* distal enhancer interaction was validated by conventional ChIP assay (Supplementary Figure 2B). Our findings highly suggest that direct transcriptional activation of *RAG1/2* by Notch1 is well conserved between mouse and human, and *cis*-elements within the -87 kb distal site in human T-ALL cells are responsible for the transcriptional activation.

### The *Rag2* Gene Locus Contains Paired Binding Sites in Response to Dimeric Notch1 Signaling

**FIGURE 3:**
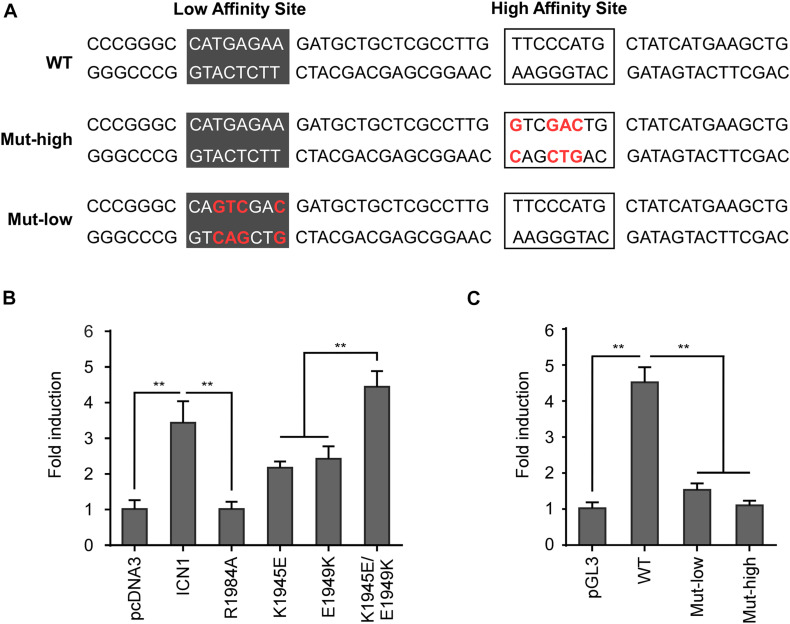
Dimeric Notch1 directly regulates *Rag2* transcription. **(A)** Schematic presentation of the predicted mouse *Rag2* SPS. The SPS, within the *Rag2* enhancer, consists of a high affinity site (marked by frames) and a low affinity site (marked by dark gray). The high and low affinity sites were mutated as indicated (red), Mut-high and Mut-low, for the following luciferase assays. **(B)** The *Rag2* SPS was constructed to luciferase reporter vector pGL3, then co-transfected with WT ICN1 or indicated mutants. Reporter activities normalized to an empty vector were determined and presented as fold induction. **(C)** The WT *Rag2* SPS or indicated mutants were constructed to luciferase reporter vector pGL3, then co-transfected with WT ICN1. Reporter activities normalized to an empty vector were determined and presented as fold induction. All of the results were shown as the mean of values from triplicate wells ± SD. Data are representative of three independent experiments. ^∗∗^*p* < 0.01.

In the microarray analysis, *Rag1* and *Rag2* were shown specifically induced by WT ICN1 but not a dimerization-disrupted mutant R1984A (Figure 1A), suggesting they are Notch1 dimer dependent. Sequence search within the −2.8-kb region revealed a dimeric Notch1 recognizable sequence-paired site (SPS). The paired site consists of two RBPJ binding sites in the head-to-head orientation, separated by 16 nucleotides (Figure 3A). The canonical RBPJ binding site CATGGGAA was considered as a high affinity site, and the other non-canonical site CATGAGAA as a low affinity site based on the previous SPS definition (Arnett et al., 2010).

To functionally validate the putative SPS, we scored various dimerization mutants in a luciferase reporter assay. The putative *Rag2* SPS was constructed upstream of a luciferase reporter and co-expressed with WT ICN1 or mutants. As expected, WT ICN1 induced a robust activation of the *Rag2* reporter gene compared with the empty vector. In contrast, R1984A resulted in little reporter activity. The other two dimerization-incompetent mutants K1945E and E1949K, impairing the intermolecular connection between Notch1 dimer, also resulted in a considerable decrease of the luciferase activity. As expected, the double mutant in *cis* K1945E/E1949K, capable of reconstituting dimeric ICN1, rescued the reporter expression (Figure 3B). These data together indicate that Notch1 dimerization is required to activate *Rag2* SPS-mediated transcription.

To directly confirm that *Rag2* SPS is a functional *cis*-element required for Notch1-mediated transcriptional activation, we mutated the high affinity binding site or the low affinity site individually to examine the *Rag2* SPS reporter gene activity. Previous reports suggest that both sites are required for cooperatively assembling dimeric complexes on DNA (Liu et al., 2010). Whereas ICN1 achieves full induction on WT SPS, mutations on either high affinity or low affinity site failed to induce the reporter gene expression (Figure 3C). Taken together, our results provide strong evidence that the identified murine *Rag2* SPS is a *bona fide* specific and functional *cis*-element, bound by Notch1 transcriptional complex and required for *Rag2* transcriptional activation. In human T-ALL cells, we noticed a highly conserved NOTCH1/RBPJ binding motif “GTGGGAA,” a canonical high affinity site, within the distal *RAG2* regulatory DNA sequence, whereas a functional SPS site remained uncertain (Supplementary Figure 2C). It seems that *cis*-elements required for *RAG1/2* transcriptional activation are not well-conserved between human and mouse, despite the analogous mechanism of Notch1-mediated transcriptional activation.

Although no SPS was revealed yet on the *Rag1* transcriptional regulatory region, both endogenous *Rag1* and *Rag2* appeared to be regulated by Notch1 dimerization (Figure 1E), raising a question why *Rag1* is sensitive to Notch1 dimer. Given *Rag1* and *Rag2* are coordinately regulated at the transcriptional level and asymmetrically disposed elements are mostly found at the 5′ of *Rag2* gene (Kuo and Schlissel, 2009), the identified SPS is most likely one of the key *cis*-elements responsible for transcriptional activation of both recombination activating genes *in vivo*.

### Enforced Dimeric Notch1 Expression in Hematopoietic Progenitors Induces Rapid *Rag1/2* Expression

**FIGURE 4:**
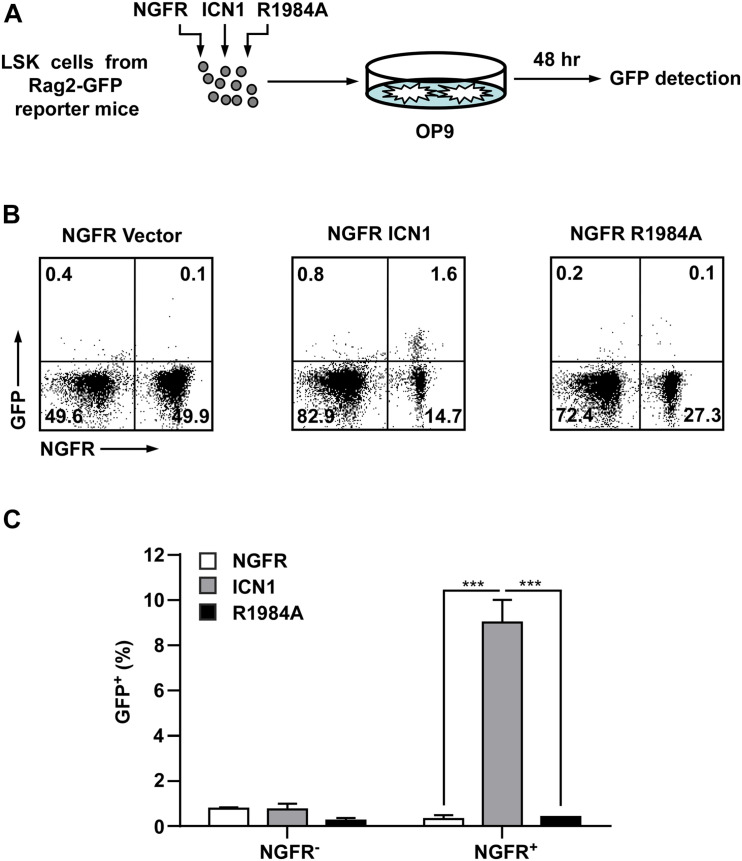
Enforced expression of dimeric Notch1 activates the *Rag2* reporter expression in hematopoietic progenitors. **(A)** Schematic presentation of the experimental procedure. GFP^–^ LSK (Lin^–^Sca^+^c-Kit^+^) cells derived from *RAG2*-GFP reporter mice (NG-BAC) were transduced with viruses expressing ICN1 or R1984A with NGFR as surrogated marker. The infected cells were co-cultured with stromal cells OP9 for 48 h and then measured the GFP^+^ percentage as an indication of *Rag2* expression. **(B)** Flow cytometry analysis of GFP and NGFR-PE expressions in hematopoietic cells recovered from OP9 culture. **(C)** A histogram presentation of GFP^+^ cell percentages in NGFR-negative or NGFR-positive populations in panel **(B)**. ***p < 0.001.

Co-expression of *Rag1* and *Rag2* during thymocyte development is essential for the assembly of functional genes encoding the TCR by recognizing and cleaving conserved recombination signal sequences located adjacent to clusters of V, D, and J gene segments (Zhang et al., 2006; Bosticardo et al., 2021). The mechanisms responsible for the lymphoid-specific and developmental stage-specific regulation of *Rag1* and *Rag2* expression are poorly understood. To determine if *Rag1* and *Rag2* were regulated by Notch1 dimerization in thymocyte development, we exploited NG-BAC mice, a *Rag2* reporter strain in which a bacterial artificial chromosome (BAC) transgene expresses a GFP in place of *Rag2*, to investigate *Rag2* transcription (Yu et al., 1999). Hematopoietic progenitors (Lin^–^Sca^+^c-Kit^+^, LSK cells) derived from NG-BAC mice were purified and transduced with retroviruses expressing the NGFR vector alone, ICN1, or the dimerization-disrupted mutant R1984A (Figure 4A). Transduced cells were subjected to *in vitro* co-culture with murine OP9 stromal cells. The *Rag2* expression, reflected by GFP fluorescence, was analyzed. As early as 48 h post-transduction, *Rag2* expression was detected in approximately 10% in ICN1 expressing (NGFR^+^) cells, whereas very few, if any, GFP^+^ cells were seen in the vector control cells (Figures 4B,C). Again, R1984A-transduced cells failed to induce *Rag2* expression in this time frame, suggesting *Rag2* expression is much more sensitive to Notch1 dimerization during thymocyte development.

**FIGURE 5:**
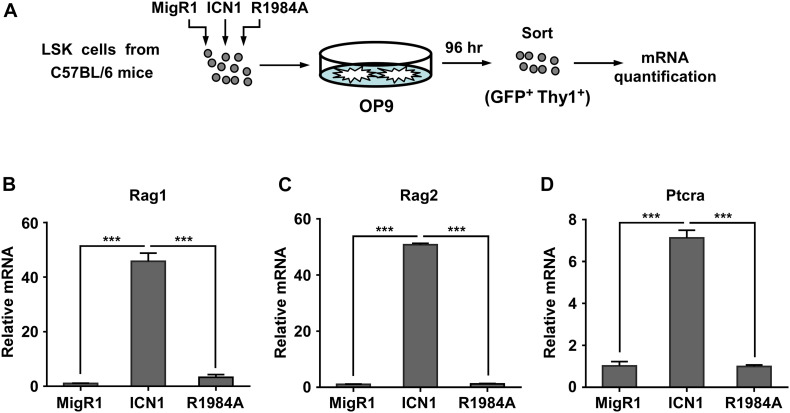
Dimeric Notch1 activates *Rag1* and *Rag2* during thymocyte development. **(A)** Schematic presentation of the experimental procedure. LSK cells derived from C57BL/6 mice were transduced with retroviruses expressing the MigR1 vector, ICN1, or R1984A with GFP as a marker. After transduction, the infected cells were co-cultured with murine OP9 stromal cells for 96 h. GFP^+^ Thy1^+^ T-lineage cells were sorted for mRNA quantification. **(B–D)** qRT-PCR analysis showing the mRNA expression of *Rag1* **(B)**, *Rag2* **(C)**, and *Ptcra* **(D)** in sorted cells. ***p < 0.001.

To further determine the role of Notch1 dimerization in the activation of *Rag1* and *Rag2* during T-cell fate specification, we purified hematopoietic stem and progenitor cells derived from C57BL/6 mice and transduced them with retroviruses expressing the MigR1 vector, ICN1, or the dimerization-disrupted mutant R1984A, then seeded them on murine OP9 stromal cells for 96 h. GFP^+^ Thy1^+^ T-lineage cells were then sorted to analyze the mRNA expression of *Rag1*/*2* and *Ptcra* (Figure 5A). As shown in Figures 5B–D, mRNA levels of *Rag1* and *Rag2* were markedly elevated in cells transduced with WT ICN1 as compared with empty vector control, while the expression of R1984A resulted in minimal induction of both genes (Figures 5B,C). As a well-characterized dimeric Notch1 target required for thymocyte development, *Ptcra* was selectively activated by WT ICN1 but not R1984A (Figure 5D). These data therefore argue the requirement of Notch1 dimerization in activating *Rag1*/*2* expression during T-cell specification.

## Discussion

The recombination activating genes 1 and 2 are crucial endonucleases to initiate V(D)J recombination in immature lymphocytes and, when overly activated, also implicated in lymphoid malignancies. Yet, transcriptional control of *Rag1* and *Rag2* is not completely understood. In this report, we demonstrate Notch1 activates *Rag1* and *Rag2* expression in both murine and human T cells. We identify a novel *cis-*element in murine T cells, which bears a dimeric Notch1 recognition site SPS and is required for Notch1-mediated transcriptional activation. Dimeric Notch1 enables the optimal expression of *Rag1* and *Rag2* in both T-ALL and T-cell progenitors. Hence, the present study manifests a prominent role of Notch1 in transcriptional activation of *Rag1* and *Rag2* in both T-cell development and leukemogenesis.

Notch1 transcriptional complex develops dimer in the nucleus on DNA sequence bearing SPS, such as the promoter of *Hes1*, a classical Notch1 target (Nam et al., 2007). Yet, the conventional SPS is not commonly detected in the genome. The hypothesis of SPS composition of one canonical site and one cryptic site was later confirmed in the enhancer of *Ptcra* and *Hes5* (Arnett et al., 2010; Liu et al., 2010). Using the matrix developed in the Blacklow Lab to predict the potential dimeric Notch1-responsive element (Arnett et al., 2010), we identified a tentative SPS proximal to the *Rag2* enhancer in murine thymocytes. Luciferase reporter assays with the site-specific mutagenesis demonstrate that the *cis*-element SPS is a *bona fide* dimeric Notch1-responsive site. Although residing on the *Rag2* transcription regulatory region, *Rag1* and *Rag2* appear dependent on dimeric Notch1 for full activation in T-ALL cells. Since inversely orientated *Rag1* and *Rag2* are coordinately regulated at the transcriptional level with major *cis*-elements identified on the *Rag2* side (Yu et al., 1999), our newly revealed SPS is most likely the DNA sequence essential for transcriptional activation of both *Rag1* and *Rag2* in murine T cells. Therefore, we not only give another example that one canonical site and one cryptic site constitute a functional SPS, but also provide a novel pair of *cis*-element (SPS) and *trans*-factor (dimeric Notch1) to dictate the transcription of *Rag1* and *Rag2*.

RAG2 has been shown as a functionally important downstream effector of Notch1 in mediating T-ALL development. T-ALL onsets were significantly delayed or impaired when ICN1 expression was enforced in the *Rag2*^–/–^ bone marrow, which was reversed upon expression of the TCRβ transgene (Allman et al., 2001; Campese et al., 2006). The outcome suggests a critical synergistic effect of Notch1 and pre-TCR signaling to initiate T-ALL. In addition, it remains possible that Notch1-induced aberrant *RAG1/2* expression creates unnecessary DSBs, eliciting inappropriate translocations or inducing DNA damages that ordinate with Notch1 to amplify oncogenic signaling and drive further neoplastic progression. For example, RAG endonuclease-elicited Notch1 truncations are revealed in almost all murine T-ALL (Tsuji et al., 2004; Ashworth et al., 2010). Likewise, in human T-ALL, there is a RAG-mediated SIL/SCL rearrangement in a Notch-dependent T-ALL line SIL-ALL, resulting in a remarkable increase of SCL (TAL1) expression (Aplan et al., 1992). We and others have shown that inhibition of Notch signaling results in *Rag1* and *Rag2* downregulation, manifesting an important correlation of Notch1 and *Rag1*/2 and also implying a role of *Rag1*/2 in Notch1-induced T-ALL (Riz et al., 2010; Kourtis et al., 2018). It is reasonable to speculate a new role of Notch1 in affecting genome instability *via* inducing *Rag1/2* expression. Another interesting finding is that depletion of *RAG1* or *RAG2* elicits T-ALL cell growth inhibition (data not shown), suggesting that these recombination activation genes may have other tumor-promoting roles in cells already transformed.

Our data also suggest that Notch1 activates *Rag2* in T-cell progenitors. Previous publications reported that inhibition of Notch1 impairs VDJβ rearrangement and TCRβ expression (Wolfer et al., 2002; Maillard et al., 2006). Although not yet demonstrated, the reduced TCRβ may result from the decreased *Rag2* expression in the absence of Notch1. Moreover, overexpression of ICN1 but not R1984A in hematopoietic progenitors led to rapid upregulation of *Rag1*/*2*, suggesting the role of dimeric Notch1 in upregulating *Rag1*/*2* in physiological conditions. However, whether Notch1 serves a primary role in activating *Rag1*/*2* expression prior to β-selection awaits further investigation. Since Notch1 expression decreases rapidly after β-selection, upregulation of *Rag1*/*2* for TCRα rearrangement is likely exerted by other transcriptional factors. Interestingly, when mice harboring Notch1 dimerization mutants were examined for the impacts of dimeric deficiency on multiorgan development, mature T-cell subsets appeared normal, despite the total number of thymocytes declined to some extent (Kobia et al., 2020). Functional validations of mature T cells in these Notch1 dimeric deficient mice are yet to be determined. Despite the paradoxical findings, we here provide compelling evidence that the transcriptional activation mediated by canonical Notch1 signaling pathway is one of the prominent mechanisms to orchestrate optimal *Rag1*/2 expression in T-cell progenitors during development and transformation.

## Materials and Methods

### Mice

C57BL/6 mice were obtained from Beijing Vital River Laboratory Animal Technology Co., Ltd., Beijing, China. NG-BAC (*RAG2*-GFP) transgenic reporter mice were as described (Yu et al., 1999). All animal experiments were performed under animal ethical regulations, and the study protocol was approved by the Institutional Animal Care and Use Committee of Wuhan University.

### Cell Culture

Murine T-ALL cell line T6E and human T-ALL cell lines KOPTK1, Jurkat, and CUTLL1 were kindly provided by Dr. Warren Pear (University of Pennsylvania, United States). The 293T cell line was purchased from the American Type Culture Collection (ATCC, Manassas, VA, United States). T-ALL cell lines were grown in complete RPMI-1640 (Gibco, Waltham, MA, United States) supplemented with 10% fetal bovine serum (FBS, Gemini Bio, West Sacramento, CA, United States), 1% penicillin/streptomycin (Hyclone, Logan, UT, United States), 1% non-essential amino acids (Gibco), 2 mM L-glutamine (Sigma, St. Louis, MO, United States), 1 mM sodium pyruvate (Sigma), and 100 μM β-mercaptoethanol (Sigma). The 293T cell was maintained in Dulbecco’s modified Eagle’s medium (DMEM, Gibco) containing 10% FBS (Gibco) and 1% penicillin/streptomycin (Hyclone). All cell lines were authenticated using short tandem repeat (STR) analysis, cultured for fewer than 2 months after resuscitation, and tested for mycoplasma contamination once a month using PCR assay.

### Reagents and Plasmids

γ-Secretase inhibitor JC-18 was a generous gift from Yue-Ming Li (Searfoss et al., 2003), and compound E was purchased from MedChemExpress (Monmouth Junction, NJ, United States). The retroviral constructs MigR1-ICN1 and MigR1-DNMAML1 (13–74)-GFP were as described (Weng et al., 2003), and MigR1-R1984A, NGFR-ICN1, and NGFR-R1984A were illustrated as previously reported (Liu et al., 2010). In the luciferase reporter assay, the murine DNA fragment containing SPS in *Rag2* transcriptional regulatory region was cloned into the pGL3 promoter vector. The forward primer is GCATATGGTACCTCGCATCCTTTTCTACTTCC and the reverse primer is ATATCTCGAGAGGAGGAGCACCACCA GTTT. The high and low affinity binding sites were subjected to mutation using QuickChange site-specific mutagenesis kit (Stratagene, San Diego, CA, United States). Primers to mutate the high affinity site are GATGCTGCTCGCCTTGGTCGACTGCTATCATGAAGCTG (forward) and CAGCTTCATGATAGCAGTCGACCAAGG CGAGCAGCATC (reverse); primers to mutate the low affinity site are GCCTATCCCCGGGCCAGTCGACGATGCTGC TCGCCTTG (forward) and CAAGGCGAGCAGCATC GTCGACTGGCCCGGGGATAGGC (reverse). All constructs were validated by DNA sequence.

### qRT-PCR

Total RNA was prepared using TRIzol (Invitrogen, Waltham, MA, United States), followed by concentration and purity determination; 1 μg of random primed total RNAs was reverse-transcribed with SuperScript II (Invitrogen) according to the instructions of the manufacturer. Primer sequences for real-time PCR were as follows: murine *Rag1*—forward, CAACGAGCACTGAAACTCCA; reverse, CCCGTCACTCTTGAAACGAT; murine *Rag2*—forward, ATTCCTGCTACCTCCCACCT; reverse, CGGAAAGCTCAT TGTTTGGT; human *RAG1*—forward, GCAGATGAGT CTGACCACGA; reverse, CTTGAAAGTCCGGAGAATGC; and human *RAG2*—forward, CAAACCCGCCATGATCTACT; reverse, TTGCTTCCTGCTGACAGATG. Primers used for *Ptcra* and *18s* rRNA were as described (Liu et al., 2010). All the primers for SYBR green detection were used at a final concentration of 0.4 μM. Real-time amplification in FAST SYBR Green Master Mix (Bio-Rad, Hercules, CA, United States) was performed on CFX Connect Real-Time PCR System (Bio-Rad). Relative expression of the mRNA was calculated by the 2^–ΔΔCt^ method and normalized to *18s* rRNA.

### Retroviral Transduction and OP9 Co-culture

High-titer retroviruses were produced as described (Chiang et al., 2008). For retroviral transduction of hematopoietic stem progenitor cells (HSPCs), LSK (Lin^–^Sca^+^c-Kit^+^) cells were purified from 6- to 8-week-old C57BL/6 or NG-BAC mice. Retroviral supernatants were added into wells coated with 20 mg/ml RetroNectin (Takara, Tokyo, Japan) and incubated for 4–6 h at 37°C before washing with PBS. Purified LSK cells were suspended in the stimulation cocktails (DMEM, 1% penicillin–streptomycin, 15% FBS, 2 mM L-glutamate, 10 ng/ml mIL-3, 10 ng/ml mIL-6, 20 ng/ml mSCF, and 20 ng/ml mFlt3L) (Liu et al., 2010) and then added to virus-bound RetroNectin-coated plates. After transduction, the infected LSK cells were plated onto fresh OP9 monolayers at 10^6^ cells/well in six-well plates. All co-cultures were performed in the presence of 1 ng/ml mIL-7 and 5 ng/ml hFlt3L within the OP9 culture medium (αMEM supplemented with 20% FBS and 1% penicillin–streptomycin) (Wong et al., 2012). For LSK cells derived from NG-BAC mice, transduced cells were analyzed by flow cytometry using PE anti-NGFR (BD Pharmingen, San Diego, CA, United States) and GFP fluorescence signals. For LSK cells derived from C57BL/6 mice, transduced T lineage cells (GFP^+^ Thy1^+^) were sorted for qRT-PCR analysis.

### Chromatin Immunoprecipitation

The ChIP assay and the subsequent quantitative PCR were performed following the protocol as described (Weng et al., 2006). Briefly, T6E or CUTLL1 cells were treated with DMSO or GSI (1 μM compound E) for 24 h prior to fixation. After fixation for 10 min in 1% paraformaldehyde at room temperature, cells were washed, lysed, and sonicated to shear DNA between 200 and 600 bp in length. Antiserum specific for Notch1 TAD domain (Wu et al., 2000) or normal rabbit IgG was used for immunoprecipitating associated DNA, which was subjected to qPCR using the SYBR system on CFX Connect Real-Time PCR System (Bio-Rad). The primers used are specified as follows: *murine hes1* promoter—forward, CGTGTCTCTTCCTCCCAT; reverse, CCAGGACCAAGGAGAGAGGT; murine *Rag2* −2.8 kb enhancer—forward, AGCTCCCCACTACCCGAGTCA; reverse, GCTGGTCCACCTTTGTTCTCTAA; *Nanog* negative control—forward, GGCTGCCTCTCCTCGCCCT; reverse, GTGCACACAGCTGGGCCTGA; and human *RAG2* −87 kb enhancer—forward, TAACTGTCAGCTGGCCACAA; reverse, TGGTCTGTCTTGCTCACCTG. Each sample was independently prepared at least two times and run in triplicate. The input DNA was defined as an aliquot of sheared chromatin resulting from crude lysate and was used to normalize the sample to the amount of chromatin added to each ChIP reaction.

### Purification of Double Negative Thymocytes

CD4^–^CD8^–^ DN thymocytes were negatively selected with anti-CD4 and anti-CD8 MACS beads according to the protocol of the manufacturer (Miltenyi Biotec, Auburn, CA, United States). After staining with antibodies against lineage (Lin) markers B220, CD19, CD11b, Gr1, CD11c, NK1.1, Ter119, CD3ε, CD8α, CD8β, TCRβ, and TCRγδ (BD Pharmingen), DN2 thymocytes (c-Kit^hi^CD25^hi^Lin^–^) were purified based on staining with anti-c-Kit and anti-CD25 antibodies (BD Pharmingen) by cell sorting on FACSAria (BD Biosciences) and analyzed on the LSR-II (BD Biosciences). DN3a (c-Kit^–/lo^CD25^hi^Lin^–^CD27^lo^) cells were purified by sorting after staining with anti-CD27 antibody (eBioscience, San Diego, CA, United States).

### Luciferase Reporter Gene Assays

Empty pcDNA3 or pcDNA3 expressing ICN1 (or indicated mutants) was transiently co-transfected in triplicate into human 293T cells with firefly luciferase pGL3-promoter vector pGL3 or driven by elements containing the *Rag2* SPS (or mutants). Cells were transfected using FuGENE 6 Transfection Reagent (Promega, Madison, WI, United States) with 0.8 μg of firefly luciferase reporter constructs, 0.1 μg of expression vectors, and 0.1 μg of Renilla luciferase expression vector. Luciferase activities were measured 24 h later using the Promega Dual Luciferase kit (Promega). Firefly luciferase activities were normalized with Renilla luciferase control values. Relative activity from the empty vector lysate was set arbitrarily to a value of 1.

### Flow Cytometry

Cells transduced with NGFR or NGFR-ICN1 and NGFR-R1984A were stained with the PE anti-NGFR (BD Pharmingen) in FACS buffer [1 × DPBS, 10 mM HEPES, 0.02% NaN_3_, 0.2% BSA (w/v)] on ice in the presence of rat and mouse IgG (Sigma-Aldrich, St. Louis, MO, United States) for 20 min, then washed and resuspended in FACS buffer. Acquisition was performed on LSRII (BD Biosciences). Dead cells and doublets were excluded based on FSC and SSC characteristics. Data were analyzed with FlowJo software (Tree Star, Ashland, OR, United States).

## Data Availability Statement

The raw data supporting the conclusions of this article will be made available by the authors, without undue reservation.

## Ethics Statement

The animal study was reviewed and approved by Institutional Animal Care and Use Committee of Wuhan University.

## Author Contributions

All authors listed have made a substantial, direct and intellectual contribution to the work, and approved it for publication.

## Conflict of Interest

The authors declare that the research was conducted in the absence of any commercial or financial relationships that could be construed as a potential conflict of interest.
