# Supplementary material for: Genome-Wide Analysis Identifies Rag1 and Rag2 as Novel Notch1 Transcriptional Targets in Thymocytes
Source: Front Cell Dev Biol. 2021 Jul 12;9:703338. doi: 10.3389/fcell.2021.703338 (PMC8311795; doi:10.3389/fcell.2021.703338)
Supplement: Supplementary file 1 [file Data_Sheet_1.docx]

**Supplementary Information**

**Genome-wide analysis identifies *Rag1* and *Rag2* as novel Notch1 transcriptional targets in thymocytes**

**Dong Y et al**

**Dong Y et al, Supplementary Figure S1**

**
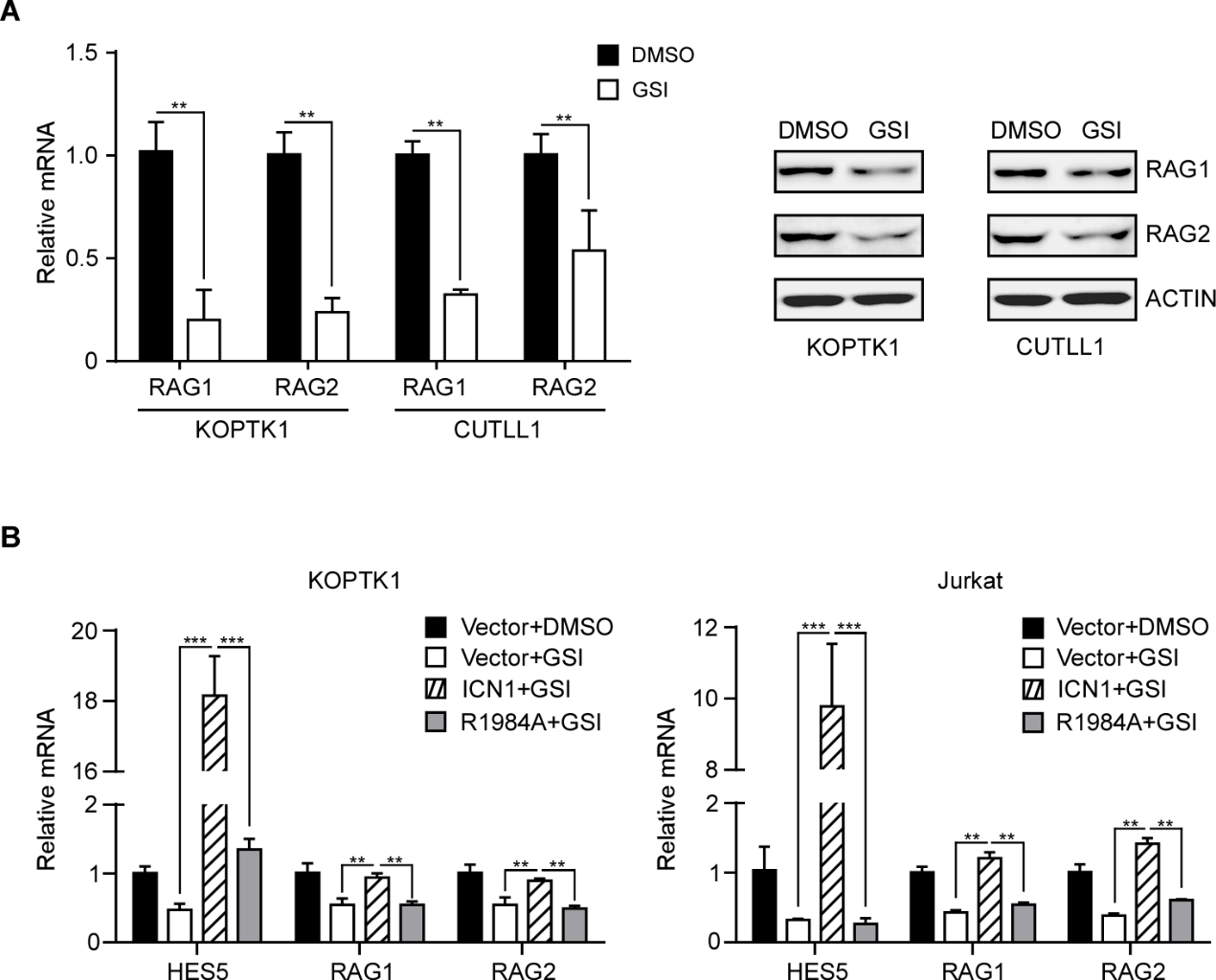
**

**Supplementary Figure S1.** Dimeric Notch1 induces *RAG1/2* expression in human T-ALL cells. **(A)** Human T-ALL KOPTK1 and CUTLL1 cells were treated with 2μM of GSI (Compound E) or DMSO for 24 h prior to total RNA extraction and reverse transcription. *RAG1/2* mRNAs were detected by real-time PCR (left). RAG1/2 proteins were assessed by immunoblots (right). **(B)** KOPTK1 (left) and Jurkat (right) cells were infected by lentiviruses expressing empty vector, WT ICN1, or R1984A mutant and subjected to GSI treatment for 24 h. *HES5*, *RAG1* and *RAG2* mRNA levels were examined by real-time PCR. Above all, the expression of *RAG1* and *RAG2* relative to *ACTIN* mRNA is shown as the mean of values from triplicate wells ± SD. Representative data from three independent experiments are shown. ***p* < 0.01, ****p* < 0.001.

**Dong Y et al, Supplementary Figure S2**


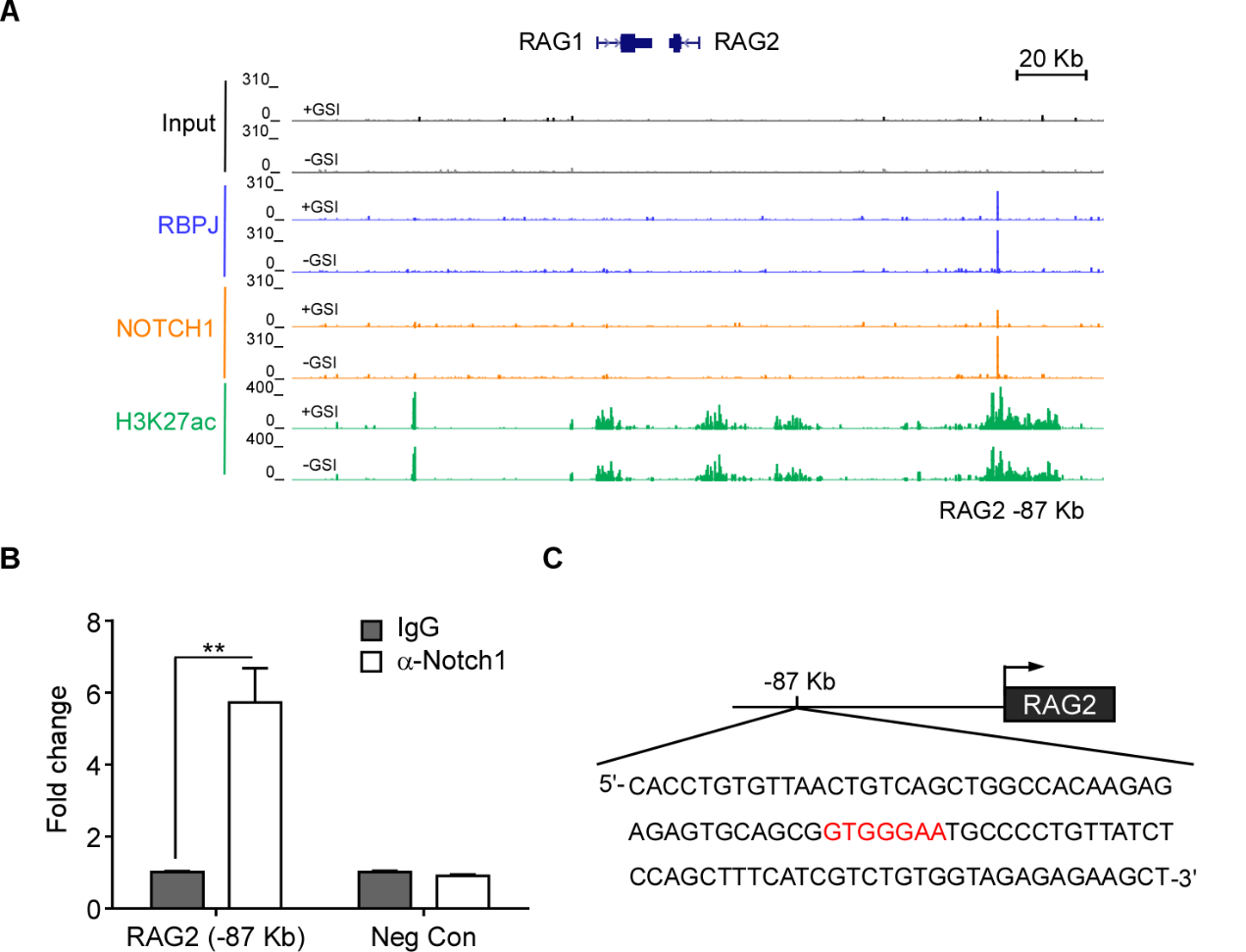


**Supplementary Figure S2.** Notch1 directly binds the *RAG*1/2 locus in human T-ALL cells. **(A)** Chromatin landscapes around the *RAG1/2* locus in CUTLL1 cells (GSE51800). The associations of nuclear Notch1, RBPJ and H3K27ac around the *RAG1/2* are shown with respect to Notch1 active (-GSI) or inactive state (+GSI). **(B)** Chromatin immunoprecipitation (ChIP) was performed on cross-linked fragmented DNAs prepared from CUTLL1 cells using the Notch1 V1744 antibody. The amount of DNA amplified from immunoprecipitated DNAs was normalized to that amplified from input DNA. A DNA sequence that is devoid of Notch1 binding site was used as the negative control (Neg Con). Each sample was prepared from at least two independent experiments and run in triplicates. ***p* < 0.01. **(C)** DNA sequences associated with the Notch1 binding region are shown and the canonical Notch1 responsive element is highlighted in red.
